# Combined impacts of sea level rise and invasive species on *Cordylanthus maritimus* (Salt Marsh Bird’s Beak) in Upper Newport Bay, California

**DOI:** 10.1371/journal.pone.0328652

**Published:** 2025-07-24

**Authors:** Hannah V. Spear, Zheyuan Zhuang, Chloe Selby, Faith Nicoll, David C. Bañuelas, Alys Arenas, Amanda Swanson, Elizabeth D. Crook

**Affiliations:** 1 Department of Earth System Science, University of California Irvine, Irvine, California, United States of America; 2 Department of Ecology and Evolutionary Biology, University of California Irvine, Irvine, California, United States of America; 3 Newport Bay Conservancy, Newport Beach, California, United States of America; 4 California Department of Fish and Wildlife, Newport Beach, California, United States of America; 5 California Invasive Plant Council, Berkeley, California, United States of America; University of Ferrara, ITALY

## Abstract

From Central California to Northern Baja California, Salt Marsh Bird’s Beak (*Cordylanthus maritimus* subsp. *Maritimus; C. maritimus*) is an annual hemiparasitic halophyte that is endemic to coastal salt marshes and is both state and federally endangered in the US and Mexico. By the year 2100, sea level is projected to rise by nearly a meter, which will decrease the biodiversity of salt marshes and lead to changes in plant community assemblages, impacting the narrow ecological niche of *C. maritimus.* Here, we address how sea level rise will impact the preferred elevation range of *C. maritimus*, and we examine how these impacts will be compounded by the presence of an invasive perennial plant species, Algerian sea lavender (*Limonium ramosissimum; L. ramossisimum*). We used LiDAR data, plant distributions, and sea level rise projections for the Upper Newport Bay Ecological Reserve to create simplified species distribution models and map current and future ranges for both species, serving as a case study for future management practices along the California Coast. In our small-scale model, the areas of these ranges and the area of overlap between both species were calculated for 2020, 2050, and 2100 under varying sea level rise scenarios. Although the overlap between the two species’ ranges currently exists, *C. maritimus* inhabits a smaller area at relatively lower elevations than *L. ramosissimum*. By the year 2100, we project *L. ramosissimum* will occupy between 200–300% more habitable area than *C. maritimus*. More than 98% of the projected habitable area for *C. maritimus* may also be suitable for *L. ramosissimum*, increasing competition between the two species and highlighting a critical need to strategically control invasive *L. ramosissimum* in Upper Newport Bay.

## Introduction

Coastal salt marshes are characterized by high productivity and biodiversity, and their limited range and unique abiotic traits increase their tendency to have specialized and adapted plant species [1,2]. These specialized plants, particularly the halophytes, are highly adapted to environments with high salinity and periodic tidal inundation, and their presence provides critical habitat for other organisms, including crustaceans, arthropods, and estuarine birds [1]. Halophytes also reduce soil salinity and improve oxygen conditions in marsh soil that would otherwise often be under anoxic conditions [2]; thus, their presence mitigates abiotic stressors for other plants in the salt marsh and creates a more habitable environment for other less salt-tolerant species [3,4]. Therefore, the presence of halophytes is critical to coastal salt marsh biodiversity and resilience, and their loss will change the composition of the plant communities, reducing species richness [1,5].

Coastal salt marshes consist of low, middle, and high marshes, creating unique habitat niches for plants and wildlife that are likely to be negatively impacted by future sea level rise (SLR). Globally, SLR is projected to increase by nearly a meter, depending on climate warming trajectories [6], which will cause a decrease in salt marsh habitats and their endemic halophytes [7–9]. This decrease in abundance will be particularly pronounced if the salt marsh remains static, with no sedimentation; it is projected that 20–60% of the world’s coastal wetlands will become inundated within the century if sedimentation is unable to keep pace with SLR [10]. Therefore, the persistence of salt marsh ecosystems depends on the ability for halophytes to vertically shift their elevation ranges to keep pace with SLR [11].

California coastal salt marshes are particularly vulnerable to SLR, due to relatively low sedimentation rates, steep topography, and or the presence of urban environments where landward transgression would be inhibited [9]. On the western coast of the US, up to 95% of salt marshes under high SLR scenarios are likely to become mudflats or inundated entirely by 2100 [9]. Endangered and endemic species in California salt marshes are therefore particularly vulnerable to SLR without upland habitat to support landward transgression [12]. Throughout coastal wetlands in California, hemiparasitic plants, in the genus *Chloropyron*, occupy a narrow elevation range, where they play a role in maintaining plant species diversity by suppressing overly dominant species [2,13]. Currently, two species of *Chloropyron* are endangered in California salt marshes and require conservation practices [14–17].

*Chloropyron maritimum subsp. maritimum* (*Cordylanthus maritimus* subsp. *maritimus*; Salt Marsh Bird’s Beak; Orobanchaceae) is a hemiparasite found primarily in the middle marsh and is protected by the federal and state endangered species acts. In Mexico, *C. maritimum* is listed as rare and threatened under Mexican Standard NOM-059-SEMARNAT-2010 [18]. The California Natural Diversity Database (CNDDB) classifies this species as a California Rare Plant Rank of 1B.2, meaning it’s listed as rare, threatened, or endangered in California and elsewhere. Historically, *C. maritimus* was widely distributed but its range has become increasingly restricted due to a variety of factors that affect dispersal including warmer temperatures, a lack of genetic diversity, extreme tide events, and urbanization [16,19,20]. As of 2020, *C. maritimus* remains extant in seven salt marshes in California, occurring between salt marshes from Morro Bay to Tijuana Estuary with 16 known locations with extinct populations [16]. Inland populations are also known to occur in San Bernardino and Los Angeles Counties, but these populations have not been recently observed [20]. Potentially, changes in climate and increasing urbanization have contributed to a shift in pollinators for *C. maritimus* from larger (Apidae and Megachilidaie) to smaller bees (Halicitdae and Colletidae) that may be less effective [21]. A myriad of factors have led to the decline of *C. maritimus* in California, including changes in available pollinators.

In recent years, the presence of invasive species has impeded efforts to restore *C. maritimus* populations. *Limonium ramosissimum* (Algerian Sea Lavender) was recently introduced from the Mediterranean and became naturalized in disturbed salt marshes throughout California [22]. In contrast to the small, realized niche of *C. maritimus*, *L. ramosissimum* has a wide distribution in both salt marshes and upland habitats, and causes a decrease in species richness [22,23]. Since *C. maritimus* relies on native plant species as hosts, the loss of native plant species richness due to invasive species will have an additional negative affect on their recovery [24]. Moreover, it remains unclear how populations of *C. maritimus* will respond in the future to both SLR and interspecific competition with *L. ramosissimum*. Since *C. maritimus* relies entirely on native host plants with no independent root system, any changes to plant hosts threatens their survival [16]. Since *L. ramosissimum* lowers the diversity of native plant species, restoration efforts using solarization (tarping), hand pulling, and are critical to protect *C. maritimus* and native hosts they rely on [24].

Here, we address the response of *C. maritimus* to the combined impacts of SLR and invasive *L. ramosissimum*. We use a simplified GIS-based model as a case study for how land managers can more effectively conserve habitats despite multiple threats to coastal environments in the future. To create habitat niche models for each species, we overlayed the distributions of *C. maritimus* and *L. ramosissimum* with a digital elevation model (DEM) in the Upper Newport Bay Ecological Reserve (UNBER) located in Orange County, California, where large distributions of *C. maritimus* populations occur [25]. Using these habitat ranges, we then used various SLR projections to model future habitable ranges for each species. Our habitat models will assist land managers with predicting the future ranges of both species and with prioritizing potential restoration sites for reintroduction of *C. maritimus*. We also provide an estimate for likely locations of invasive *L. ramosissimum*, both now and in the future, so that removal efforts can be maximized with limited resources. Using this case study, these cost-effective measures can be broadly applied to other coastal regions, species, and habitats for future conservation efforts. By using elevation we provide a broad range of future occurrences to allow future planning to realize the full extent of populations compared to more narrow specific models.

## Methods

### Study area

Upper Newport Bay Ecological Reserve (UNBER) is part of Southern California’s largest estuarine complex in Orange County and encompasses approximately 304 hectares of endangered wetland, salt marsh, and transitional habitat (117°52’55.03“W 33°38’56.152”N). Designated an Ecological Reserve in 1975, UNBER has a long history of disturbance that includes salt production and dredging operations and rapid urbanization of the surrounding watershed.

The current topography of the terrestrial environment was established by the removal 1.9 million m^3^ of sediment during the 1980’s. Today UNBER has a maximum elevation of 11.75m, with most of the salt marsh consisting of lower elevations, with a mean of 1.97m above sea level (NAD83 Datum). The changes in elevation support distinct habitats that include mudflats and lower, middle, and upper marshes. Native plant species that serve as hosts plants for *C. maritimus* in Southern California and UNBER include *Batis martima*, *Distichlis spicata, Distichlis littoralis, Frankenia salina*, *Limonium californicum*, *Jaumea carnosa*, and *Salicornia pacifica* [16]. Additionally, in the UNBER *C. maritimus* occurs with dense populations of *S. pacifica* [26]. Prior evidence shows that overabundance of *S. pacifica* is not ideal for *C. maritimus* [15]. While there is ample information collected by the Newport Bay Conservancy and its partner agency Tidal Influence on the occurrence of *C. maritimus*, a study analyzing the co-occurrences with native plants has yet to be undertaken as conducted for other members in the genera.

There is limited information on the introduction of *L. ramosissimum* in UNBER and in California salt marshes in general; however, removal efforts began in 2016 with hand pulling and solarization treatments [24]. Additionally, Asian sea lavender (*Limonium otolepis*) and European sea lavender (*Limonium duriusculum*) occur in low abundances in UNBER. Here, we focus on *L. ramosissimum,* as this is the species that is present in greatest abundances, poses the greatest threat to native species, and already co-occurs with *C. maritimus* within UNBER.

### Niche models

Distributions of *C. maritimus* and *L. ramosissimum* in UNBER were established between 2019 and 2020 through ground surveys. The surveys for *C. maritimus* were conducted between June and July 2019 by Tidal Influence LLC using a Trimble Geo 7X Handheld. Polygons were obtained in the field using the streaming feature on the GPS device, where GPS points were recorded when the occurrence of *C. maritimus* was less than 1m^2^. All salt marshes were searched within the Reserve and a total of 0.004 km^2^ were recorded. Similarly, ground surveys for *L. ramosissimum* were conducted between June 2019 and March 2020 using a Garmin GLO 2 Bluetooth GPS Receiver. All spatial data was stored in a geodatabase and archived using a polygon or point. LiDAR data of UNBER was used for the high-resolution digital elevation values in our models. LiDAR data was compiled over 108 flights between October 2009 and August 2011, and covers 6,775km^2^ of California coast (https://www.fisheries.noaa.gov/inport/item/48166). Water bodies, including tidal water bodies such as UNBER were hydro flattened to mean sea level. The LiDAR data used the NAD83 datum and were converted to a digital elevation model prior to use and clipped for the extent of UNBER.

Plant occurrence data were overlaid onto the LiDAR data in ArcGIS (ESRI), and a preferred range in elevation (based on observed occurrences) for each plant species was obtained. This preferred elevation range was then used in conjunction with the LiDAR data to map the overall potential habitable areas of each species using elevation alone. That is, we calculated the total area within the habitable elevation range for each plant species using the LiDAR data, and the habitable areas reported are maximum ranges based on the observational data obtained in the field (the models do not point to exact locations where the species are found).

Other factors that are often included in species distribution models, such as vegetation alliance, soil, and precipitation data, were excluded from our models due to the relatively small study area, homogeneity of the study site, and strong influence of elevation over species distribution. Elevation is the primary factor for the distribution of these species because the salinity of the soil and level of inundation is dependent on proximity to mean sea level [9]. Previous research has also shown that annual rainfall can determine the extent of the *C. maritimus* [16]. By centering the modeling effort on elevation, we can examine the total habitat suitability without other factors such as rainfall and vegetation alliance. This approach produces fewer limiting factors in our projection models and represents a best-case scenario for *C. maritimus*. As *L. ramosissimum* is a niche generalist and has been found in high densities outside of the reserve, using elevation data alone is a conservative estimate for the range of this invasive species. These species distribution models, based on preferred elevation above mean sea level, were then used to model how the total habitable area of each species may change due to rising sea levels. In dong so our models could also determine what future influence *L. ramosissimum* could have on *C. maritimus* via a overlap in potential habitat.

Previous studies have noted that sedimentation rates are low for UNBER [9] and that the uncertainty for the sea level rise projections is greater than the historical sedimentation rates of 2.5 mm per year [9]. Low sediment accretion combined with a probable increase in erosion rates with SLR make the future of sedimentation uncertain. We therefore include both estimates, with and without sedimentation, in our models to provide a range of estimates by the year 2050 and 2100.

### Sea level scenarios and models

Sea level rise projections were obtained from the Intergovernmental Panel on Climate Change (IPCC) Sixth Assessment Report [6]. The sea level rise projections for UNBER varies based on different climate change scenarios and range from a 0.18− 0.23m increase by 2050, and from a 0.38− 0.77m increase by 2100. These scenarios are dependent on various representative concentration pathways (RCPs) projected for the coming century [6]. Representative concentration pathways are based on projected CO_2_ emissions by the end of the 21^st^ century and correspond to changes of radiative forcing (i.e., heat) that will lead to varying rates of SLR. Projected SLR for 2050 and 2100 for the five different scenarios (intermediate low, intermediate, intermediate high, high, and extreme) are summarized in Table 1. For this study, we focus on Intermediate, High, and Extreme SLR projections.

**Table 1:** Representative concentration pathway scenarios and associated SLR projections.

Representative Concentration Pathway	Sea Level Rise by 2050 (m)	Sea Level Rise by 2100 (m)
Intermediate Low	0.18	0.38
Intermediate	0.19	0.44
Intermediate High	0.21	0.56
High	0.22	0.68
Extreme	0.23	0.77

Representative Concentration Pathways (RCPs) as defined by the Intergovernmental Panel on Climate Change (IPCC) and their corresponding sea level rise projections for the Upper Newport Bay. For this study, we focus on Intermediate, High, and Extreme SLR projections.

Two different species distribution models for UNBER were created. The first model assumed no sediment accretion, and the second assumed historical sedimentation rates of 2.5 mm/year. Both models assumed that landward transgression of each species was possible, and that vertical migration would occur in response to rising sea levels. That is, both the lower and upper bounds of the habitable range were adjusted using the LiDAR data according to the projected SLR scenario. Each model was generated for the three scenarios, for 2050 and 2100, yielding 12 total models for *C. maritimus* and 12 total models for *L. ramosissimum*. All future ranges were compared to present day ranges. We then overlaid the model predictions for the future ranges of the two species and determined the areas of overlap allowing us to predict how the habitable range of *C. maritimus* may be impacted by *L. ramosissimum* in conjunction with SLR. As a final step, to determine areas for restoration prioritization for *C. maritimus*, we overlayed the 6 models for 2050 and the 6 models for 2100 to determine hotspot regions where *C. maritimus* is predicted to have a maximum habitable range in response to SLR.

## Results

### C. maritimus

**Fig 1:**
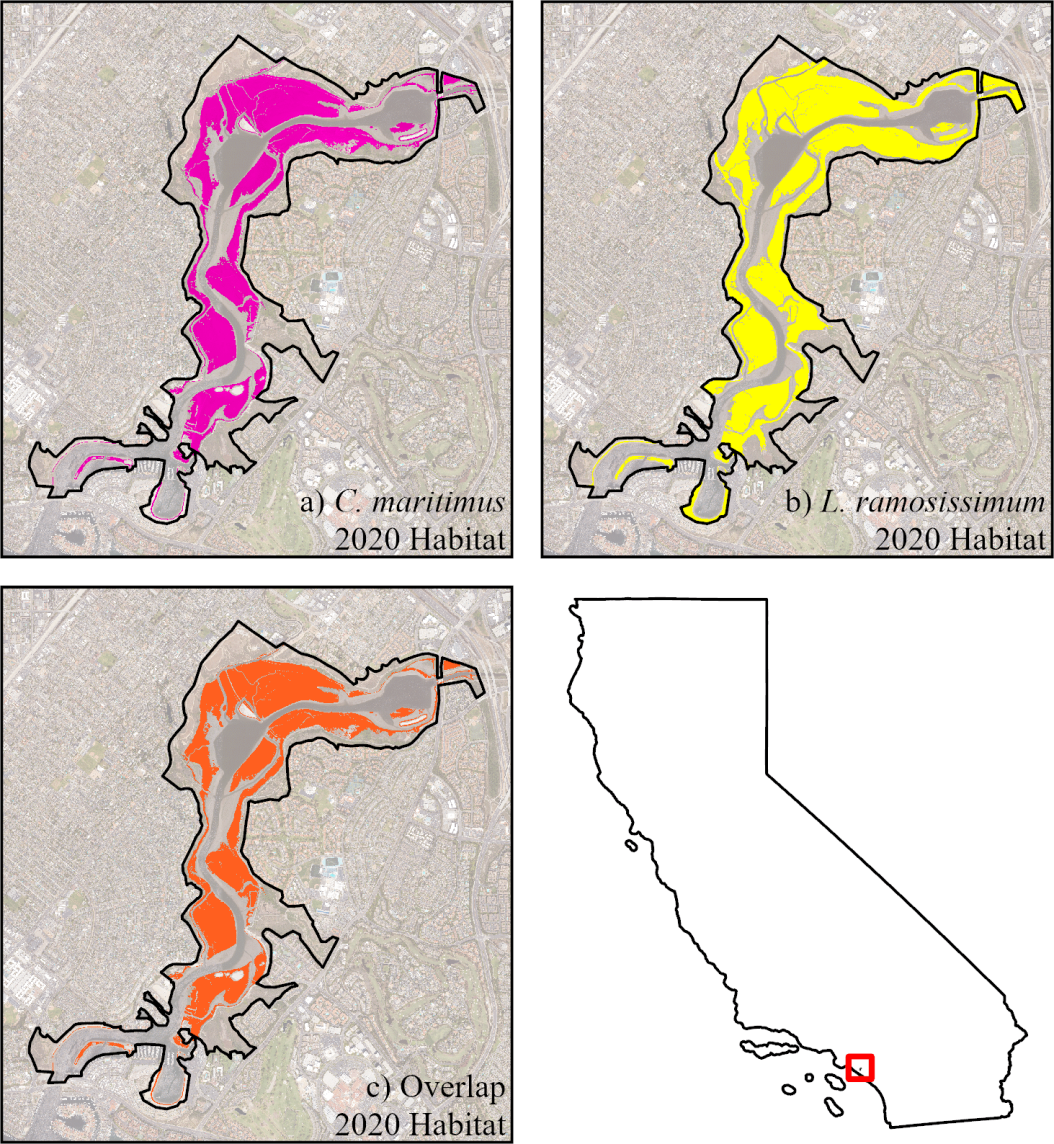
Modeled distribution range of current (2020) *C. maritimus and L. ramosissimum* in the UNBER. a) shows the habitable range of *C. maritimus* (pink), b) shows the habitable range of *L. ramosissimum* (yellow), and c) shows the overlapping ranges of the two species (orange). These distributions represent all possible locations the plants could be found based on their known ranges above sea level within UNBER, but do not show actual occurrence of the species. The location of UNBER is provided along the coast in the inset map of California. A public domain basemap was downloaded from the NOAA Satellite Maps application: https://coast.noaa.gov/digitalcoast/data/highresortho.html.

Based on distribution data obtained from field observations, *C. maritimus* inhabits a narrow range in elevation, with all observations occurring between 0.35m and 0.69m above mean sea level (MSL); mean elevation was 0.57m above MSL. The current habitable range of *C. maritimus* was modeled by selecting all elevation values that fall between 0.35−0.69m (Fig 1a). Fig 1a shows the entire possible habitable range of *C. maritimus* based solely on elevation and does not necessarily indicate the presence of the species in those locations. Thus, the model shows a much larger habitat niche availability for *C. maritimus* than is actually observed within UNBER. Under present conditions (2020), the total habitable range for *C. maritimus* in UNBER is 1.46km^2^, though the realized niche for the species is much more limited where the total habited area estimated at 0.004km^2^ based on occurrences in 2020. Our models are therefore meant to be a first-order indicator of all possible locations for the species, and do not consider other biotic and abiotic factors that might limit their presence.

**Fig 2:**
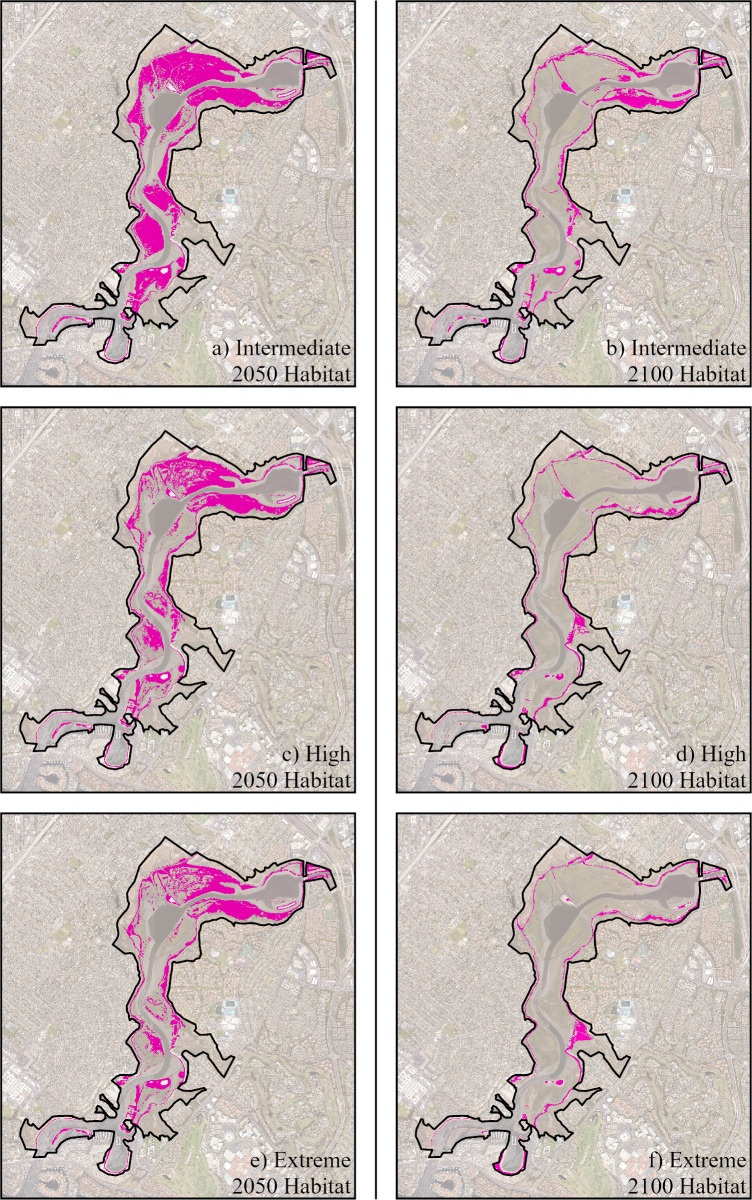
Projected *C. maritimus* habitable range in 2050. Habitable ranges for three SLR scenarios (Intermediate, High, and Extreme) by 2050 and 2100. These models assume sedimentation accretion of 2.6 mm/year. Highlighted regions show areas of priority for land managers by 2050 and 2100 based on these elevations falling within the habitable range of the species with a given SLR scenario. A public domain basemap was downloaded from the NOAA Satellite Maps application: https://coast.noaa.gov/digitalcoast/data/highresortho.html.

Projected *C. maritimus* habitable ranges calculated for each of the SLR scenarios assume that it will respond to rising sea level by shifting its habitable range to higher elevations, so both the lower and upper bound for the species were shifted (Fig 2). That is, we modeled the likely scenario in which *C. maritimus* would not be able to survive unless it’s ideal habitat range of 0.35−0.69m above MSL were realized. Two models were used: the first assumes historical sedimentation rates of 2.6 mm/year were realized (Fig 2) and the second did not include sedimentation to account for probable increased erosion rates with SLR (S1 Fig). Using both models, we can provide an upper and a lower value estimate for the projected habitable range of the species by 2050 and 2100 (Table 2). We predict that the potential does exist for *C. maritimus* to migrate landward in response to SLR, as the preferred elevation range of the species remains available in UNBER with current SLR projections. However, as SLR continues to inundate the current shoreline, the species could have a reduction in habitable area of up to 92% from 2020 values by the year 2100 from 1.46km^2^ to 0.13km^2^ (Fig 2, Table 2).

**Table 2:** Total habitable area projections (*C. maritimus*) for the year 2050 and 2100.

Representative Concentration Pathway	Habitable Area 2050 (km^2^), no sedimentation	Habitable Area 2050 (km^2^), with sedimentation	Habitable Area 2100 (km^2^), no sedimentation	Habitable Area 2100 (km^2^), with sedimentation
Intermediate	0.42	1.08	0.23	0.35
High	0.37	0.69	0.15	0.22
Extreme	0.36	0.59	0.13	0.19

Projections are based on current (2020) observed habitable elevation range preferences (0.35m to 0.69m above MSL). Both model scenarios are provided, with and without sedimentation. Models assume range shifts are possible, where the species was able to respond to rising sea level by adjusting its vertical elevation range above sea level. For example, a sea level rise of 0.5m would result in landward (vertical) migration of the species by 0.5m, for a new habitat range of 0.85m to 1.09m above MSL.

**Fig 3:**
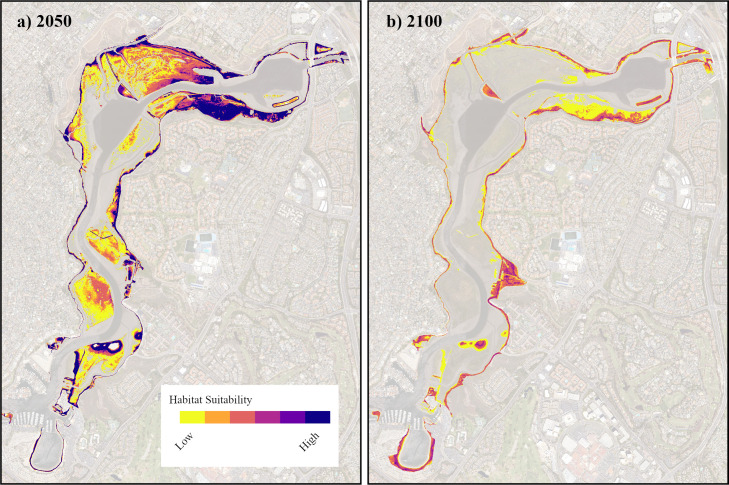
Priority Areas for Conservation of *C. maritimus* in 2050 and 2100. Habitable range projections for all three SLR scenarios (Intermediate, High, and Extreme), both with sedimentation and without sedimentation, were overlayed in 2050 (a) and 2100 (b), for a total of 6 possible layers in each image. Hotspot areas indicate where conservation should be prioritized in the future for *C. maritimus* based on the number of models that predict a particular area falls within the future habitable range of the species. A public domain basemap was downloaded from the NOAA Satellite Maps application: https://coast.noaa.gov/digitalcoast/data/highresortho.html.

Because of the uncertainty in our models concerning the unknown sediment accretion and erosion rates in response to SLR, a hotspot analysis for the projected ranges of *C. maritimus* was produced by overlapping each of the models for the year 2050 and 2100. That is, this analysis included the 3 predicted ranges with sedimentation (Intermediate, High, and Extreme) and the 3 projected ranges without sedimentation, for the year 2050 and again for the year 2100 (Fig 3). The resulting analysis shows the likelihood of a given area to be within the habitable range of *C. maritimus* in 2050 and 2100.

### L. ramosissimum

The current habitable range (year 2020) for *L. ramosissimum* within UNBER was found to be between 0.36 and 2.05m, with a mean elevation of 0.72m above mean sea level. The total current habitable range (2.01km^2^) for this invasive species was modeled by selecting all elevation values within this preferred elevation range within the Reserve (Fig 1). This current total habitable range is approximately 30% larger for *L. ramosissimum* than for *C. maritimus*. In addition, the current habitable range of *L. ramosissimum* occupies the entire habitable range of *C. maritimus* and is likely to be present in much higher concentrations throughout this range.

**Fig 4:**
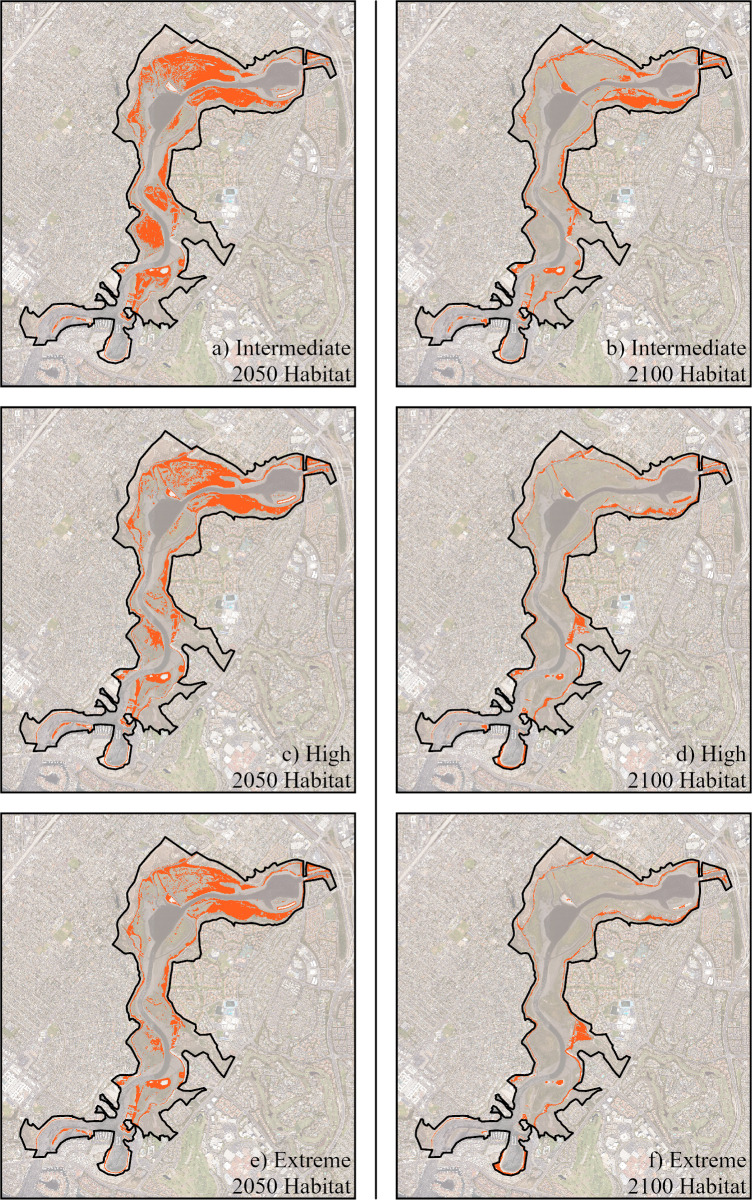
Projected *L. ramosissimum* habitable range in 2050 and 2100 that overlaps with the potential range of *C. maritimus.* Habitable range overlaps are modeled assuming landward migration is possible for both species and that sedimentation occurred at an annual rate of 2.6 mm/year. Three SLR scenarios are modeled: Intermediate, High, and Extreme. A public domain basemap was downloaded from the NOAA Satellite Maps application: https://coast.noaa.gov/digitalcoast/data/highresortho.html.

Because of this overlapping range between the species, complete encroachment of *C. maritimus* by *L. ramosissimum* in the future could be possible, as landward transgression of the species will be hindered by the presence of the invasive plant. Using the niche models to calculate total habitable area, we project that *L. ramosissimum* will be able to inhabit an increasingly larger area than *C. maritimus* through time (Table 3). Estimates for 2050 scenarios vary widely, with *L. ramosissimum* occupying anywhere from 1.35 to 2 times larger an area than *C. maritimus* (S2 and S3 Figs). For the 2100 scenarios, *L. ramosissimum* has approximately 2–3 times the total available area for landward migration compared to *C. maritimus*. When comparing habitable ranges, we found that across all SLR scenarios for both 2050 and 2100, 98% or more of the *C. maritimus* habitat falls within the range of *L. ramosissimum* (Fig 4).

**Table 3:** Total habitable area projections (*L. ramosissimum*) for the year 2050 and 2100.

Representative Concentration Pathway	Habitable Area 2050 (km^2^), no sedimentation	Habitable Area 2050 (km^2^), with sedimentation	Habitable Area 2100 (km^2^), no sedimentation	Habitable Area 2100 (km^2^), with sedimentation
Intermediate	0.84	1.47	0.57	0.76
High	0.78	1.06	0.45	0.55
Extreme	0.76	0.99	0.43	0.50

Projections are based on current (2020) observed habitable elevation range preferences (0.36m to 2.05m above MSL). Both models’ scenarios are provided, with and without range shifts. Models with range shifts were derived from an approach where the species was able to respond to rising sea level by adjusting its elevation range (i.e., a sea level rise of 0.5 m resulted in landward (vertical) migration of the species by 0.5m).

## Discussion

The current potential habitable area of *C. maritimus* is limited by its very narrow preferred elevation range above MSL, which is very likely to be inundated by SLR within the next century. Its habitat within UNBER is confounded by the presence of *L. ramosissimum*, which has a nearly complete overlapping habitable range. While the habitable ranges of both species in the marsh will be substantially reduced by the year 2100 because of SLR, our projections also indicate that *L. ramosissimum* will be able to inhabit an increasingly larger portion of the *C. maritimus* niche through time. Additionally, *L. ramosissimum* has a higher maximum elevation range within the marsh, reaching higher elevations above MSL, likely hindering any potential landward transgression of *C. maritimus*. Therefore, *L. ramosissimum* not only outcompetes *C. maritimus* for habitable space, but it also will likely prevent the species from occupying higher elevations in response to SLR.

These models suggest that mitigation of *C. maritimus* is possible within UNBER so long as the preferred future habitat range of the species is conserved (0.35−0.69m above MSL). Our models therefore show precise locations where the removal of *L. ramosissimum* should be prioritized to enable the landward migration of *C. maritimus* in the very near future. We also suggest that mitigation is now critical, as beyond 2050, SLR is likely to have negative and severe consequences to the very narrow preferred habitat range of *C. maritimus*.

There are several factors that may limit the use of our simple models or make future predictions more complex. There are many other physical and biological parameters that contribute to the small realized niche of *C. maritimus* within UNBER which are not factored into our models, there is a range of uncertainty for SLR projections [6], and future sedimentation rates may change due to increasing erosion caused by SLR and land use practices within the marsh (including dredging and boat use). We attempt to limit these uncertainties by estimating impacts of both low and high-end SLR projections, thus encapsulating a wide range of predictions. We also use the highest known sedimentation rates for these models. Additionally, as the high and extreme SLR projection scenarios are more likely than an intermediate scenario based on our current CO_2_ emissions trajectories, these maps represent a best-case scenario for *C. maritimus* and should inform land management practices in response to future climate change.

Our models predict the locations within the marsh where conservation efforts for the removal of *L. ramosissimum* should be prioritized, given limited resources and labor. That is, our models are not designed to predict precisely where *C. maritimus* will appear in the future: rather, these simplified models are meant to provide the probable locations where *C. maritimus* has the greatest likelihood of survival given future SLR projections. The need to conserve land at slightly higher elevations where *C. maritimus* may need to find refuge in the future is imperative. Given that some amount of habitat loss is inevitable in the next 30–70 years, regardless of projection scenario, removal of *L. ramosissimum* should also be targeted in regions where it is likely to infringe upon the future range of *C. maritimus*. Therefore, these maps highlight areas that may be critical for the survival of *C. maritimus* beyond the 21^st^ century and may need to be considered when managing habitat to promote *C. maritimus* populations in the future. This is particularly important within UNBER because restoration and monitoring efforts are limited by funding to support staff and materials, and therefore pinpointing locations where efforts can be maximized for future populations is particularly useful.

Our maps indicate that *L. ramosissimum* may invade much of the *C. maritimus* habitat in the future. This is likely due to several factors that give *L. ramosissimum* a competitive edge over *C. maritimus*, including its current prevalence at higher elevations above MSL, meaning it is already established in the critical zones of landward migration for *C. maritimus* in the future. Unlike *C. maritimus*, seeds of *L. ramosissimum* disperse by hydrochory, floating on fluctuating tides and currents [24,27]. Once established in salt marshes, *L. ramosissimum* forms dense monocultures that decrease available hosts for *C. maritimus* [19,23]. In other coastal salt marshes across Californian, *L. ramosissimum* has also spread rapidly, especially throughout the San Francisco Bay Estuary [23]. Given its competitive advantages, *L. ramosissimum* will likely eradicate *C. maritimus* from UNBER and California within the next 30 years without conservation efforts to control its spread. These conservation efforts may also include outplanting with native species following removal of *L. ramosissimum*. This will prevent recurrence and support the migration of native marsh plants that form a vegetation alliance with *C. maritimus*.

Reduction in *C. maritimus* habitats through time may have critical cascading effects on other marsh species, including endangered migrating bird species. The invasion of *L. ramosissimum* in UNBER has impacted several federal and state listed birds who rely on a diverse array of native vegetation for nesting [28]. Therefore, conservation of endangered flora and fauna in California must focus on removing invasive species like *L. ramosissimum*. It is therefore of critical concern to conserve the remaining marsh habitat suitable to *C. maritimus* to protect the species from future effects of SLR and competition of invasive species. Our maps highlight regions where removal of *L. ramosissimum* should be prioritized, and they provide targeted sites for the future restoration of *C. maritimus* within UNBER. Indeed, efforts are currently underway to remove *L. ramosissimum* and dredge critical habitats within UNBER in preparation for SLR. These efforts include hand-pulling treatments, which are costly and time-intensive. Understanding the potential migration *C. maritimus* allows for the concentration of efforts in potentially critical habitat locations of the future.

Since *L. ramosissimum* can be found throughout salt marshes in California, where several species of *Chloropyron* occur, the results have relevance for beyond the UNBER [23]. Salt marsh ecosystems in the Pacific Coast, especially California, are susceptible to habitat loss because of the lack of lateral migration due to development [9]. Even more difficult, *Chloropyron molle* subsp. *molle* (Soft Bird’s Beak) in the San Francisco Bay is dispersal limited which may inhibit lateral migration [15]. In Central California, SLR model projections also estimate a 40% reduction in habitable area for *C. maritimus* [29]. In Southern California, based on precipitation and tidal influence, it is predicted that *C. maritimus* will have “boom years” where the collection of seeds is important for future planting efforts during “bust” years [16]. The results of our study also align with previous findings for the Carpinteria Marsh, where *C. maritimus* occurrences in high marshes are most susceptible to SLR [30]. Given that invasive species like *L. ramosissimum* are widespread from Northern to Southern California and overlap with *C. maritimus* and *C. molle* habitats, targeted treatments and restoration efforts are crucial [23,24]. Moreover, *C. maritimus* serves as a key indicator of a resilient salt marsh ecosystem, relying on a diverse assemblage of native halophytes [16,19,20]. Therefore, conserving *C. maritimus* is not only critical for its own intrinsic value but also to preserve the broader native plant community. Conversely, the presence of *L. ramosissimum* is often associated with degraded and disturbed salt marshes with low plant diversity [23,24]. While it is established that Southern California’s steep topography and lack of inward migration for salt marshes will ‘squeeze out’ *C. maritimus*,’ this study indicates *L. ramosissimum* as a rising threat [31]. Our findings therefore highlight the urgent need to incorporate *L. ramosissimum* into SLR projections, prioritizing management interventions in areas where its spread is likely to further impact *C. maritimus* populations.

On a much broader scale, these models serve as a case study for how similar projections can be made given relatively few resources and limited funding for land managers in wetlands throughout California and beyond. For example, monitoring precise locations of a targeted species every year can be costly, time consuming, and unfeasible in practice for an agency. Our models show that the current precise locations of plants within a California wetland can be used to understand how the range of the population may be impacted by SLR over the next 100 years. For the case of *C. maritimus* and *L. ramosissimum* in Southern California, restoration projects such as the Big Canyon Project have already started that are based on sea level rise projections [32]. Globally, such restoration projects to control invasive species populations while conserving endangered species is critical. Our work shows that simplified habitable range models such as this can be made for a targeted species given a digital elevation model, species distribution information, and SLR projections for a given coastline.

## Supporting information

S1 Fig Projected *C. maritimus* habitable range in 2050, without sedimentation. Habitable ranges for three SLR scenarios (Intermediate, High, and Extreme) by 2050 and 2100. These models assume no sedimentation accretion. Highlighted regions show areas of priority for land managers by 2050 and 2100 based on these elevations falling within the habitable range of the species with a given SLR scenario. A public domain basemap was downloaded from the NOAA Satellite Maps application: https://coast.noaa.gov/digitalcoast/data/highresortho.html.
(TIF)

S2 Fig Projected *L. ramosissimum* habitable range in 2050, with sedimentation. Habitable ranges for three SLR scenarios (Intermediate, High, and Extreme) by 2050 and 2100. These models assume sedimentation accretion of 2.6 mm/year. Highlighted regions show areas of likely *L. ramosissimum* migration by 2050 and 2100 based on these elevations falling within the habitable range of the species with a given SLR scenario. A public domain basemap was downloaded from the NOAA Satellite Maps application: https://coast.noaa.gov/digitalcoast/data/highresortho.html.
(TIF)

S3 Fig Projected *L. ramosissimum* habitable range in 2050, without sedimentation. Habitable ranges for three SLR scenarios (Intermediate, High, and Extreme) by 2050 and 2100. These models assume no sediment accretion. Highlighted regions show areas of likely *L. ramosissimum* migration by 2050 and 2100 based on these elevations falling within the habitable range of the species with a given SLR scenario. A public domain basemap was downloaded from the NOAA Satellite Maps application: https://coast.noaa.gov/digitalcoast/data/highresortho.html.
(TIF)
